# Out-of-Plane Compression Behaviour of Aluminum Alloy Large-Scale Super-Stub Honeycomb Cellular Structures

**DOI:** 10.3390/ma16031241

**Published:** 2023-01-31

**Authors:** Song Lin, Meini Yuan, Bing Zhao, Beibei Li

**Affiliations:** 1 College of Mechatronics Engineering, North University of China, Taiyuan 038507, China; 2 AVIC Manufacturing Technology Institute, Beijing 100015, China; 3 School of Civil Engineering, Hefei University of Technology, Hefei 230009, China

**Keywords:** aluminium alloy honeycomb, friction stir weld, experiment, finite element (FE) analysis, local buckling design method

## Abstract

The out-of-plane compression behaviour of 6061-T6 aluminum alloy super-stub honeycomb cellular structures without and with friction stir welding (FSW) facesheets are presented in this paper. A total of twelve axially compressed experiments on large-scale specimens, six with square hollow section (SHS) cores and six with hexagonal hollow section (HHS) cores, were conducted, with failure modes, ultimate resistances and axial load-end shortening curves analysed. The accuracy of finite element (FE) models was validated in accordance with test results. The numerical data obtained from extensive parametric analyses combined with test data were subsequently used to evaluate the applicability of existing design rules in Chinese, European and American aluminium alloy specifications. The results showed that the three specifications generally yielded very conservative predictions for the out-of-plane compression resistances of SHS and HHS super-stub honeycomb cores without and with FSW facesheets by about 30–37%. Design recommendations on the cross-section effective thickness are finally proposed and shown to provide much more accurate and consistent predictions than current design methods. The research results are beneficial to the application and development of large-scale super-stub honeycomb structures in structural engineering, such as the helicopter landing platforms, the base of fluid and gas tanks and ship decks.

## 1. Introduction

Aluminium alloy honeycomb structures generally consist of cellular cores and facesheets and have been extensively applied in the aerospace, transport and medical industries, due to their favorable properties of high energy-absorbing rate, great strength-to-weight ratio, good corrosion resistance and high specific strength and stiffness [1,2,3]. The mechanical properties of this kind of structure, including the out-of-plane and in-plane compression resistances, bending behaviour and energy dissipation capacities subjected to impact or quasi-static loadings, have been experimentally and numerically investigated by many researchers. The damage and failure response of 3003-H19 aluminium alloy honeycomb cores glued with 1100-H14 aluminium alloy facesheets under low-velocity impact were experimentally studied and then numerically modeled according to the work conducted by Foo et al. [4]. Three-point bending tests on carbon-fiber and aluminum alloy honeycomb sandwich structures reinforced by 6060-T5 aluminum alloy grid were carried out by Shi et al. [5] to analyse their flexural strength and energy absorption capacity. Hussein et al. [6] experimentally studied the crushing response of 6060-T5 aluminum alloy square tubes filled with 5052-H39 aluminium alloy honeycomb core or polyurethane foam subjected to out-of-plane quasi-static compressed loads. The damage process, buckling strength and energy absorption capacity of aluminum alloy honeycomb cores glued with and without facesheets under out-of-plane compression were experimentally, numerically and theoretically performed by Dai et al. [7]. Gong et al. [8] investigated the localised impact resistances of aluminium alloy honeycomb structures with positive, zero and negative Poisson’s ratios by numerical analysis, with a dual-wall honeycomb proposed.

However, the sizes of aluminum alloy honeycomb cells mentioned above are generally very small, with cell thickness and cell size of less than 0.1 mm and 6 mm, respectively, leading to the cells being unable to sustain great out-of-plane compression loading. It can be expected that the out-of-plane compression resistances of honeycomb cores with relatively large dimensions of cells, such as cell thickness and size of up to 5 mm and 100 mm, respectively, are tremendously improved compared with small cells. Therefore, aluminum alloy large-scale honeycomb structures have great application potential in helicopter landing platforms, the base of fluid and gas tanks, ship decks, etc. Many researchers studied the local buckling behaviour and axial compression resistances of stub columns with large sizes, including the AA6060 aluminum alloy square hollow sections (SHS) [9], 6060-T6, 6082-T6 and 6061-T6 aluminum alloy SHS and rectangular hollow sections (RHS) [10], 6063-T5 and 6061-T6 aluminum alloy circular hollow sections (CHS) [11], 6061-T6 and 6063-T5 aluminum alloy SHS and RHS with internal cross stiffeners [12], 6061-T6 and 6063-T5 aluminum alloy H sections [13], 7A04-T6 aluminum alloy SHS and RHS [14] and 7075-T6 aluminum alloy H sections [15]. However, existing studies mainly focused on the axial compression behaviour of aluminum alloy stub columns with single RHS, SHS, CHS and H sections and the height of three times the depth of their sections, and few investigations on the out-of-plane compression behaviour of aluminum alloy large-scale super-stub honeycomb cores are reported, which imposes a great restriction on the application and development of large-scale super-stub honeycomb structures in structural engineering.

The out-of-plane compression behaviour of aluminum alloy large-scale super-stub honeycomb structures was experimentally and numerically investigated in this paper. A total of twelve specimens, six with SHS cores and six with hexagonal hollow section (HHS) cores, were axially compressed, and the accuracy of finite element (FE) models on the compression resistances and failure modes were validated in accordance with test results. The numerical data obtained from extensive parametric analyses combined with test data were used to evaluate the applicability of existing design rules in Chinese standard (GB 50429-2007) [16], European specification (EN 1999-1-1:2007) [17] and American aluminium design manual (AADM-2015) [18]. Design recommendations on the cross-section effective thickness were finally proposed and verified by test and numerical data.

## 2. Experimental Study

### 2.1. Testing Specimens

**Figure 1:**
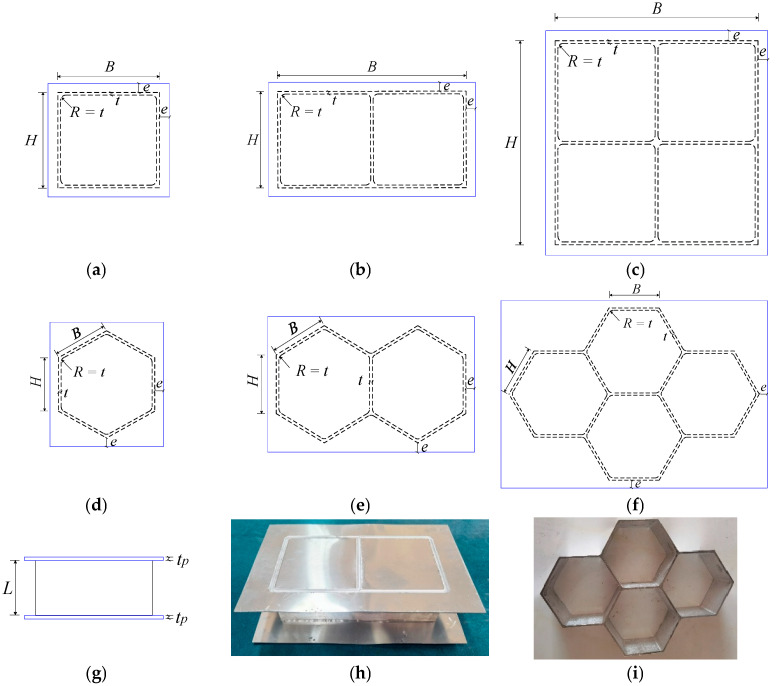
Configurations of honeycomb specimens: (**a**) Single SHS cell with FSW facesheets (top view); (**b**) Double SHS cells with FSW facesheets (top view); (**c**) Four SHS cells with FSW facesheets (top view); (**d**) Single HHS cell with FSW facesheets (top view); (**e**) Double HHS cells with FSW facesheets (top view); (**f**) Four HHS cells with FSW facesheets (top view); (**g**) Elevation view; (**h**) Photo of double SHS cells with FSW facesheets; (**i**) Photo of four HHS cells.

**Figure 2:**
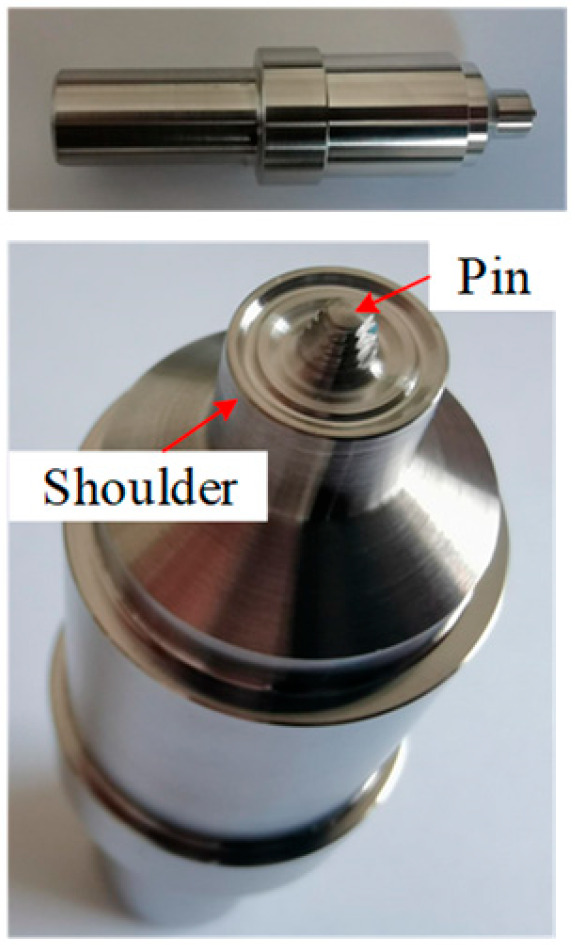
FSW tool.

**Figure 3:**
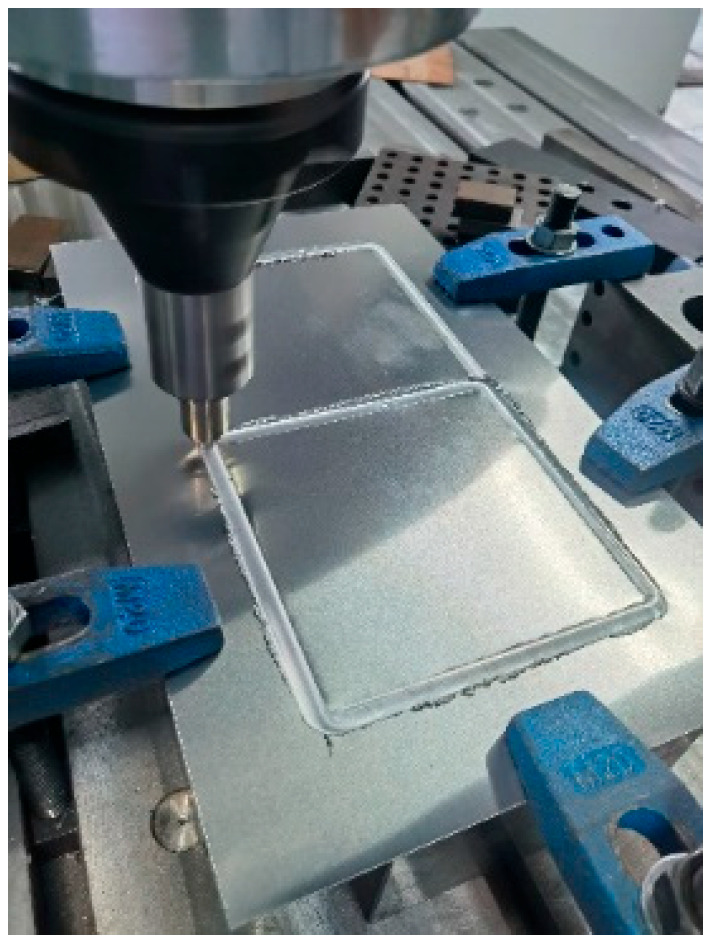
Welding process and clamping device.

An experimental programme was performed to investigate the out-of-plane compression resistances of 6061-T6 aluminium alloy large-scale square hollow sections (SHS) and hexagon hollow section (HHS) honeycomb cores without and with friction stir welding (FSW) facesheets. A total of twelve specimens were considered, including non-welded specimens (S180-5-1, S180-5-2, S180-5-4, H180-5-1, H180-5-2, H180-5-4) and specimens with FSW facesheets (S180-5-1-FSW, S180-5-2-FSW, S180-5-4-FSW, H180-5-1-FSW, H180-5-2-FSW, H180-5-4-FSW), as illustrated in Figure 1. The first letter of the identifier of each specimen denotes the SHS or HHS, and the subsequent three Arabic numerals signify the outer width, thickness and the number of SHS or HHS cells, respectively. In addition, “FSW” added in the last position of the identifier means that the specimen was friction stir welded with facesheets. Note that all specimens were milled from a 100 mm thick 6061-T6 aluminium alloy plate, so the height of all specimens is *L* = 100 mm. In order to evaluate the feasibility of the novel FSW for fixing facesheets on specimens to provide an alternative to replace the traditional adhesive method, 2 mm thick facesheets were welded to a 5 mm thick honeycomb structure by the FSW, as shown in Figure 2. The relative density of square and hexagonal structures is 5.6% and 10.8%, respectively. The FSW tool had a concave shoulder of 10 mm in diameter and a key pin with a thread taper profile and three grooves. The pin length, pin root diameter and pin tip diameter are 2.5 mm, 3.2 mm and 2 mm, respectively. The clamping device and welding process are presented in Figure 3. The tool rotational speed, transverse speed, tilt angle and penetration depth of the tool shoulder were set as 1050 r/min, 80 mm/min, 2.0° and 0.1 mm, respectively, after a series of trial weldings. On the basis of the slenderness limits set out in EN 1999-1-1:2007 [17] and GB 50429-2007 [16], non-welded SHS and HHS honeycomb specimens are respectively classified as slender and non-slender sections, while SHS and HHS honeycomb specimens with FSW facesheets are all within the slender class. The measured dimensions of all honeycomb specimens are listed in Table 1, where *B*, *H*, *t* and *t_p_* are illustrated in Figure 1, *A*_g_ is the gross section area of a specimen and *e* = 20 mm.

### 2.2. Material Properties

**Figure 4:**
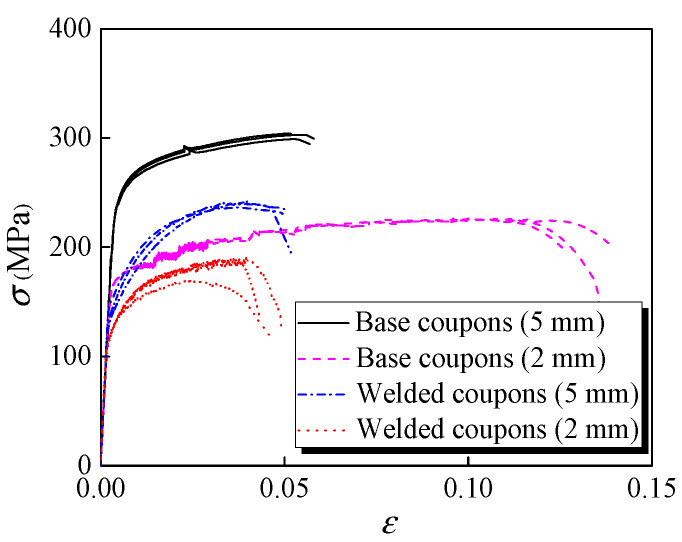
Measured stress-strain curves.

**Figure 5:**
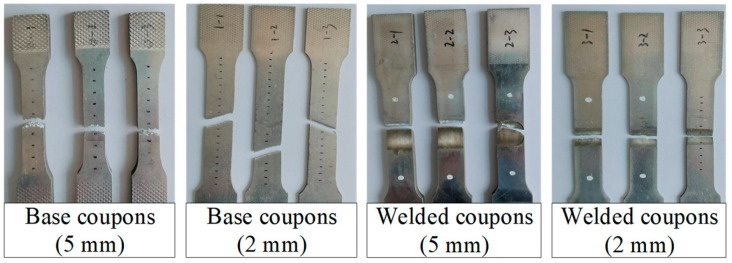
Failure modes of coupons.

Prior to the out-of-plane compression tests, tensile coupon tests of the base and friction stir welded aluminium alloys were conducted to determine the material properties. The tensile coupons of base aluminium alloys were extracted from the cross-sections along the height of specimens, while welded tensile coupons were obtained from butt joints perpendicular to the weld direction, given that it is difficult to quantify accurately the mechanical properties of welded T-joint. The stress-strain curves of base and welded coupons with 5 mm and 2 mm thicknesses are plotted in Figure 4, with failed coupons shown in Figure 5.

**Figure 6:**
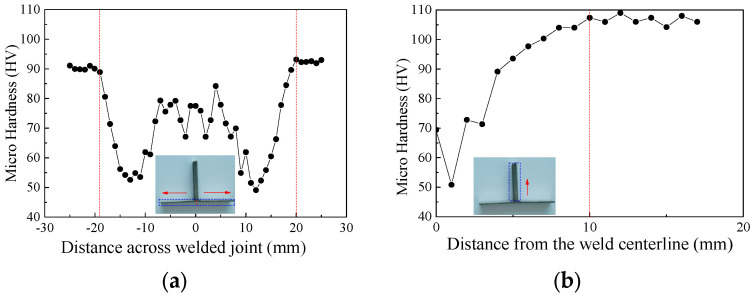
Microhardness distribution of the FSW T-joint: (**a**) Skin of T-joint; (**b**) Stringer of T-joint.

The specific elastic modulus (*E*), 0.2% proof strength (*f*_0.2_), ultimate strength (*f*_u_) and ultimate strain (*ε*_u_) of each coupon are summarised in Table 2. It can be seen that the strength reduction factors were 0.61 and 0.76 in terms of *f*_0.2_, respectively, which were less than those in terms of *f*_u_ (of 0.79 and 0.81) and the mean strength reduction factor could be determined as 0.74. The Ramberg–Osgood model (*ε* = *σ*/*E* + 0.002(*σ*/*f*_0.2_)*^n^*) was adopted to match the measured stress-strain curves and the fitted exponents (*n*) are listed in Table 2. The results show that the exponent (*n*) of the welded aluminium alloys was significantly smaller than those of base ones, indicating that a relatively obvious strain-hardening effect occurred in welded joints. Additionally, microhardness tests of the welded T-joint were also carried out to obtain the heat-affected zone (HAZ) of the FSW T-joint used in specimens, as depicted in Figure 6. The width of HAZ was found to be about 20 mm for the 2 mm thick facesheets and 10 mm for the 5 mm thick specimens.

### 2.3. Out-of-Plane Compression Tests

**Figure 7:**
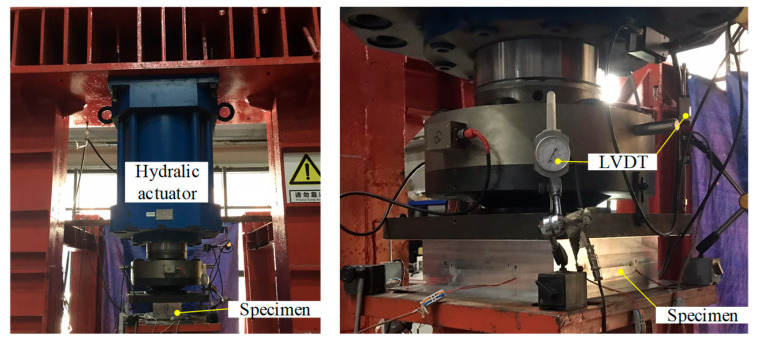
Testing setup.

Out-of-plane compression tests on twelve 6061-T6 aluminium alloy large-scale SHS and HHS super-stub honeycomb cores without and with FSW facesheets were performed to obtain their behavior and resistance. The testing setup is illustrated in Figure 7. A 5000 kN hydraulic actuator mounted on the reaction frame was used to generate designated loads on each specimen with a constant loading rate of 0.05 mm/min. Four linear variable differential transformers (LVDTs) were respectively arranged at the four corners of each specimen to record the end shortening.

**Figure 8:**
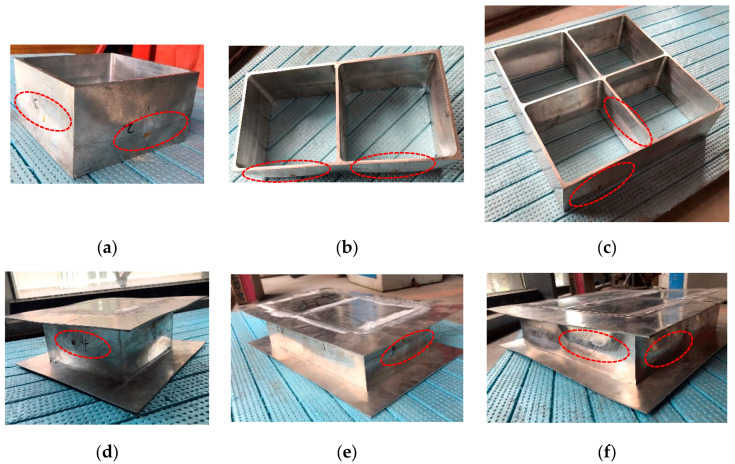
Failure modes of SHS honeycomb specimens: (**a**) Single SHS cell; (**b**) Double SHS cells; (**c**) Four SHS cells; (**d**) Single SHS cell with FSW facesheets; (**e**) Double SHS cells with FSW facesheets; (**f**) Four SHS cells with FSW facesheets.

**Figure 9:**
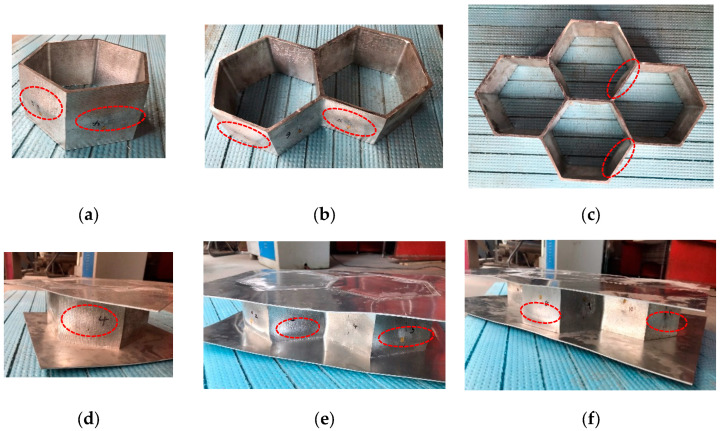
Failure modes of HHS honeycomb specimens: (**a**) Single HHS cell; (**b**) Double HHS cells; (**c**) Four HHS cells; (**d**) Single HHS cell with FSW facesheets; (**e**) Double HHS cells with FSW facesheets; (**f**) Four HHS cells with FSW facesheets.

**Figure 10:**
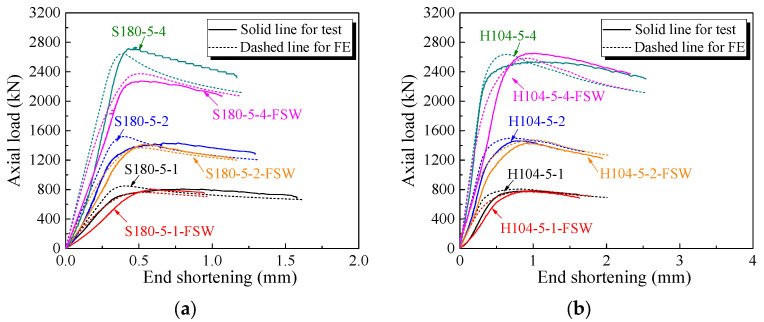
Axial load versus end shortening curves of honeycomb specimens: (**a**) SHS specimens; (**b**) HHS specimens.

All specimens failed by local buckling deformation before and after cross-section yielding, as shown in Figure 8 and Figure 9 for the SHS and HHS honeycomb specimens, respectively. The axial load-end shortening curves are plotted in Figure 10a,b for the SHS and HHS honeycomb specimens, respectively. The axial compression resistances of all specimens are summarised in Table 3. The normalised resistances (*N*_exp_/(*A*_g_*f*_0.2_)), where *N*_exp_ is the experimental resistances of specimens, of non-welded and welded SHS honeycomb specimens were all less than unity. While the normalised resistances of non-welded and welded HHS honeycomb specimens were in the range of 1.02–1.08, which was not consistent with the classification of slender sections for HHS honeycomb specimens with transverse welds, indicating the potentially little influence of FSW on section resistances. The ratios of the out-of-plane compression resistances of honeycomb specimens with FSW facesheets to those of non-welded honeycomb specimens were 0.83–1.0 for SHS specimens and 0.98–1.05 for HHS specimens, respectively, showing the feasibility of FSW technology for fixing facesheets on two ends of honeycomb cores to form an integrated structure. Although the cross-sectional areas of the square specimens are larger than those of the hexagonal specimens by 11–17%, the differences in out-of-plane compression resistances between the square and hexagonal specimens are within 5%, mainly due to the fact that the local buckling deformation occurred before cross-section yielding for square specimens.

## 3. Numerical Simulation

### 3.1. Numerical Modelling

**Figure 11:**
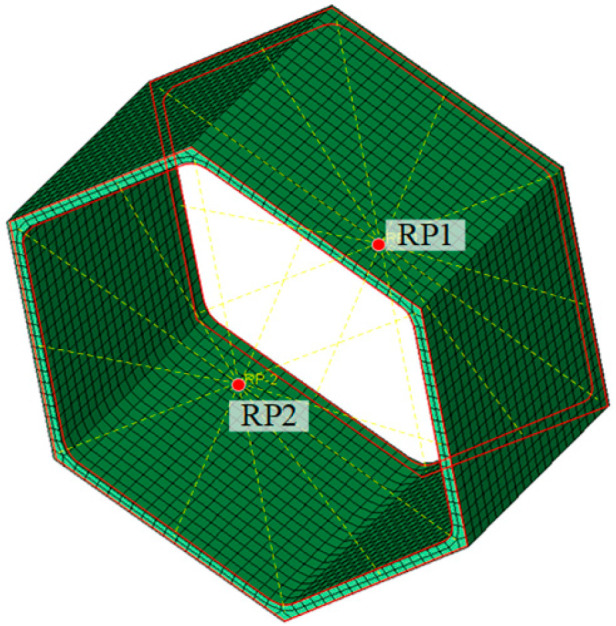
Numerical model of honeycomb specimens.

ABAQUS software [19] was employed to establish finite element (FE) modes of all honeycomb specimens, as shown in Figure 11. The true stress *σ*_true_ = *σ*(1 + *ε*) and logarithmic plastic strain $\varepsilon_{\mathrm{true}}^{\mathrm{pl}}$ = ln(1 + *ε*) − *σ*_true_/*E* were inputted into the material model, where *σ* and *ε* are the engineering stress and strain, respectively, and taken as the average measured results of three repeated coupons from Table 2. Note that the aluminium alloy materials within the HAZ should take the strength reduction effect into account by using the coupon results of welded joints. The solid element C3D8R rather than the shell element was applied for all honeycomb specimens to accurately simulate the variable thickness of cross-sections at corner regions. The mesh sizes of 5 mm for honeycomb cores and 4 mm for facesheets were selected based on a mesh sensitivity study, which could achieve the expected accuracy with acceptable computational efficiency. Regarding the boundary conditions, the non-welded honeycomb ends or outer faces of the facesheets of the welded honeycomb specimens were respectively coupled to reference points RP1 and RP2, with all degrees of freedom of two reference points restrained except the longitudinal translation at RP1. The facesheets were tied to the honeycomb ends for welded specimens. The first local buckling mode obtained from elastic Eigenvalue buckling analysis was taken as the initial local geometric imperfection mode, with amplitude of *w*_0_ = 0.033(*f*_0.2_/*σ*_cr_)*t* [20], where *σ*_cr_ is the elastic buckling stress of a four-side simple supporting thin plate under uniform compression loads and determined as *σ*_cr_ = 4*π*^2^*E*/[12(1 − *v*^2^)(*b*/*t*)^2^], herein *v* is the Poisson’s ratio.

### 3.2. Validation of FE Models

**Figure 12:**
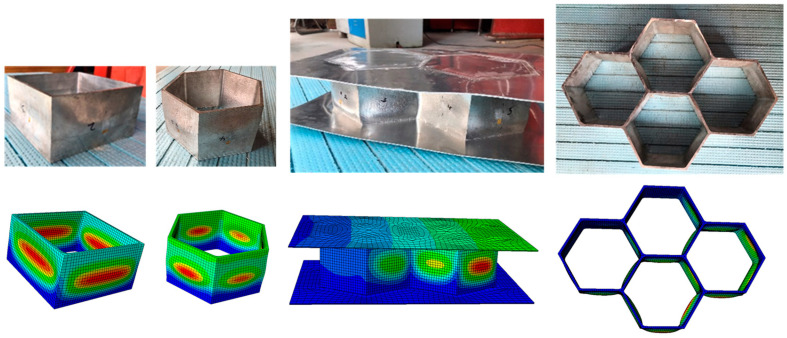
Comparisons between numerical and experimental failure modes of honeycomb specimens.

The local buckling deformation before and after cross-section yielding for respective HHS and SHS honeycomb specimens was observed from FE models, which were consistent with test results, as illustrated in Figure 12. The axial load-end shortening curves obtained from the FE models and experiments were plotted in Figure 10, with ratios of numerical resistances (*N*_FE_) to those experimental ones (*N*_exp_) listed in Table 3. The average ratio of *N*_FE_/*N*_exp_ and the corresponding coefficient of variation (COV) were 1.01 and 0.037, respectively, indicating that the FE models were capable of accurately predicting the out-of-plane compression resistances of non-welded and welded 6061-T6 aluminium alloy large-scale super-stub honeycomb specimens with single, double and four SHS or HHS cells.

### 3.3. Parametric Analysis

Upon validated FE models, extensive parametric analyses were conducted to enrich the data pool on the out-of-plane compression behaviour of non-welded and welded honeycomb specimens with single, double and four SHS or HHS cells. A total of 33 specimens for each case, including a single SHS cell, double SHS cells, four SHS cells, single SHS cell with facesheets, double SHS cells with facesheets and four SHS cells with facesheets, were developed, resulting in 198 specimens for SHS honeycomb specimens. The width-to-thickness ratio of the SHS honeycomb specimens varied from 16.0 to 80.0 and the cross-sections were classified as Class 3 and Class 4, as listed in Table 4. A similar approach was used to generate HHS honeycomb specimens, with a total of 192 HHS specimens and width-to-thickness ratios of 16.05–80.05 shown in Table 5. Note that the height of the FE models was kept at 100 mm to investigate the out-of-plane compression behaviour of super-stub honeycomb specimens. The cell thickness and facesheet thickness remained constant at 5 mm and 2 mm, respectively, while the outer sections varied. The material properties, boundary conditions, element type, mesh size and initial local geometric imperfection of the FE models were the same as those in Section 3.1. The parametric analysis results showed that the out-of-plane compression resistances of welded SHS and HHS super-stub honeycomb structures were 0.89–1.0 times those of non-welded SHS and HHS super-stub honeycomb specimens, indicating that the facesheets can be effectively fixed on the end of SHS and HHS super-stub honeycomb cores to form sandwich configuration by FSW without failure occurring at the welded zone.

## 4. Out-of-Plane Compression Design

In this section, the design provisions set out in Chinese standard (GB 50429-2007), European standard (EN 1999-1-1: 2007) and the American aluminium design manual (AADM-2015) were introduced and selected to predict the out-of-plane compression resistances of SHS and HHS honeycomb cores without and with FSW facesheets based on the experimental and numerical results. Moreover, a revised Chinese design method was finally suggested.

### 4.1. GB 50429-2007

The axial compression resistance of stub columns can be calculated by the product of the cross-sectional area and the nominal yield strength, as given by Equation (1),

(1) $$N_{\mathrm{GB}}=\left\{\begin{array}{l} A_{g}f_{0.2}/\gamma_{\mathrm{GB}}\;\;\;b/t\leq \xi_{\mathrm{GB}}\sqrt{240/f_{0.2}}\\ A_{e}f_{0.2}/\gamma_{\mathrm{GB}}\;\;\;b/t>\xi_{\mathrm{GB}}\sqrt{240/f_{0.2}} \end{array}\right.$$

where *γ*_GB_ is the resistance factor and taken as 1.2; *A*_g_ and *A*_e_ are the gross and effective cross-section area, respectively; *ξ*_GB_ = 21.5 for SHS and HHS stub columns without welds, while *ξ*_GB_ = 17 for SHS and HHS stub columns with welds. It can be seen that the specimen cross-section was first categorised as non-slender or slender according to the limit of the width-to-thickness ratio of $\xi_{\mathrm{GB}}\sqrt{240/f_{0.2}}\;$. The thickness reduction is used to determine the effective area for slender sections to consider the negative influence of local buckling and welds on the axial compression resistance, as given by Equations (2) and (3),

(2) $$\frac{t_{e}}{t}=\frac{\alpha_{1}}{\lambda_{p}}-\frac{0.22\alpha_{2}}{\lambda_{p}^{2}}\leq 1$$

(3) $$t_{e,\mathrm{haz}}=\rho_{\mathrm{haz}}t$$

where *λ*_p_ is the non-dimensional slenderness of the plate and determined as $\lambda_{p}=\sqrt{f_{0.2}/\sigma_{\mathrm{cr}}}$; *α*_1_ and *α*_2_ are the constants and both taken as 1.0 for SHS and HHS stub columns without welds and 0.9 for SHS and HHS stub columns with welds. *ρ*_haz_ is the strength reduction factor within the HAZ due to welds and is taken as 0.5 for 6061-T6 aluminium alloys using metal inert gas (MIG) welding and tungsten inert gas (TIG) welding. The final effective cross-section thickness within the HAZ of welded specimens subjected to uniform compression load is taken as the lesser of that corresponding to the reduced thickness (*t*_e_) and that corresponding to the reduced thickness in the HAZ (*t*_e,haz_).

### 4.2. EN 1999-1-1: 2007

Compared with GB 50429-2007, similar design provisions of the axial compression resistance of stub columns are provided in EN 1999-1-1: 2007, as given by Equations (4)–(6),

(4) $$N_{\mathrm{EC}9}=\left\{\begin{array}{l} A_{g}f_{0.2}/\gamma_{\mathrm{EC}9}\;\;b/t\leq \xi_{\mathrm{EC}9}\sqrt{250/f_{0.2}}\\ A_{e}f_{0.2}/\gamma_{\mathrm{EC}9}\;\;b/t>\xi_{\mathrm{EC}9}\sqrt{250/f_{0.2}} \end{array}\right.$$

(5) $$\frac{t_{e}}{t}=\frac{C_{1}}{(b/t)/\sqrt{250/f_{0.2}}}-\frac{C_{2}}{{\left[(b/t)/\sqrt{250/f_{0.2}}\right]}^{2}}$$

(6) $$t_{e,\mathrm{haz}}=\rho_{0.2,\mathrm{haz}}t$$

where *γ*_EC9_ is the resistance factor and equal to 1.1; *ξ*_EC9_ = 22 for SHS and HHS stub columns without welds, while *ξ*_EC9_ = 18 for SHS and HHS stub columns with welds; *C*_1_ and *C*_2_ are the constants and codified as 32 and 220, respectively, for SHS and HHS stub columns without welds, while they are 29 and 198, respectively, for SHS and HHS stub columns with welds; *ρ*_0.2,haz_ is the nominal yield strength reduction factor, and equal to 0.48 for 6061-T6 aluminium alloys due to MIG and TIG welding. Note that the effective thickness of cross-sections (*t*_e_) shall be used to calculate the effective cross-section area for the Class 4 (slender) cross-section without welds. The final effective cross-section thickness within HAZ of welded specimens under uniform compression load is the same as that of Chinese code, namely min(*t*_e,haz_, *t*_e_).

### 4.3. The American Aluminum Design Manual (AADM-2015)

The design rules of axial compression resistance of stub columns in AADM-2015 are related to the compressive critical stress and gross cross-section area, as determined by Equation (7),

(7) $$N_{\mathrm{AADM}}=\phi_{c}f_{c}A_{g}$$

where *ϕ*_c_ is the resistance factor and equal to 0.9; *f*_c_ is the compressive critical stress, corresponding to three limit states of yielding, inelastic buckling and post-buckling, which is determined by Equation (8),

(8) $$f_{c}=\left\{\begin{array}{l} f_{0.2}\;\;\;\;\;\;\;\;\;\;\;b/t\leq \lambda_{1}\\ B_{p}-1.6D_{p}b/t\;\;\;\;\lambda_{1}<b/t\leq \lambda_{2}\\ k_{2}\sqrt{B_{p}E}/(1.6b/t)\;\;\;\;b/t>\lambda_{2} \end{array}\right.$$

where *λ*_1_ = (*B*_p_ − *f*_0.2_)/(1.6*D*_p_), *λ*_2_ = (*k*_1_*B*_p_)/(1.6*D*_p_), *B*_p_ and *D*_p_ are the buckling constants, *B*_p_ = *f*_0.2_[1 + (*f*_0.2_/(1500*κ*))^1/3^] and *D*_p_ = (*B*_p_/10)(*B*_p_/*E*)^1/2^ for SHS and HHS stub columns without welds, while *B*_p_ = *f*_0.2_[1 + (*f*_0.2_/(440*κ*))^1/3^] and *D*_p_ = (*B*_p_/20)(6*B*_p_/*E*)^1/2^ for SHS and HHS stub columns with welds; *k*_2_ is the post-buckling constant and equal to 2.27 and 2.04 for non-welded and welded specimens.

### 4.4. Evaluation of Current Codified Design Methods

**Figure 13:**
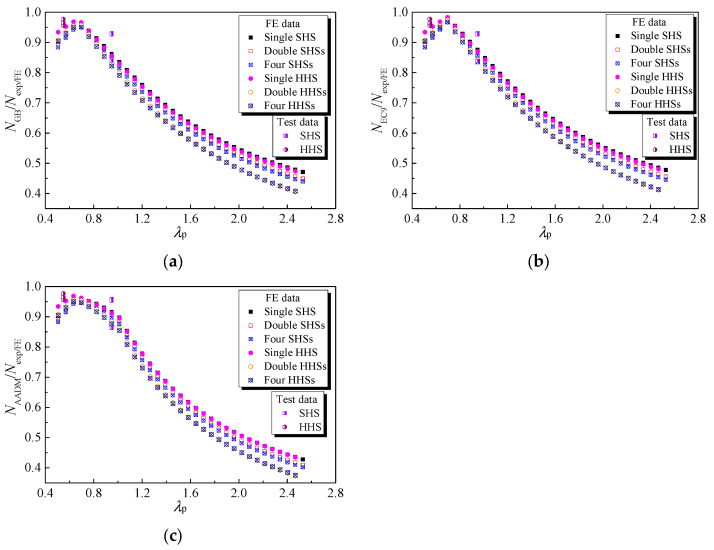
Comparisons of test and FE results with GB 50429, EC9 and AADM for non-welded honeycomb specimens: (**a**) GB 50429; (**b**) EC9; (**c**) AADM.

**Figure 14:**
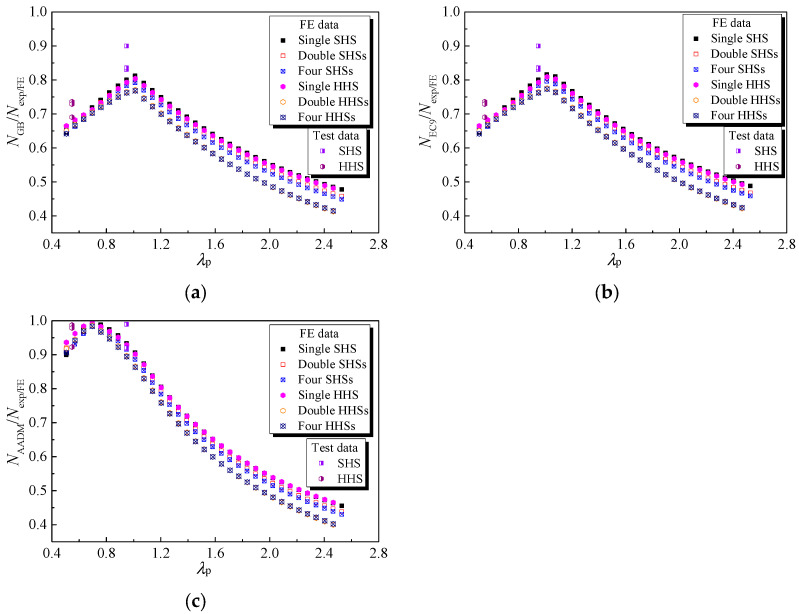
Comparisons of test and FE results with GB 50429, EC9 and AADM for honeycomb specimens with FSW facesheets: (**a**) GB 50429; (**b**) EC9; (**c**) AADM.

The out-of-plane compression resistances of 6061-T6 aluminium alloy large-scale SHS and HHS super-stub honeycomb cores without FSW facesheets predicted from GB 50429-2007, EN 1999-1-1: 2007 and AADM-2015, normalised by the test and FE data, are plotted against the non-dimensional slenderness of the plate in Figure 13. Note that the resistance factors mentioned in the three specifications shall be unity to determine their nominal strengths. The comparison results of large-scale SHS and HHS super-stub honeycomb cores with FSW facesheets are illustrated in Figure 14. With regard to large-scale SHS and HHS super-stub honeycomb cores without FSW facesheets, it was observed that the ratio of predicted resistance to test/FE data increased while *λ*_p_ was less than 0.7, while it gradually decreased as *λ*_p_ went beyond this limit; moreover, they were all less than unity, indicating the compression resistance predictions from the three specifications were consistently conservative, especially for honeycomb specimens with larger *λ*_p_. Similar trends were also found for large-scale SHS and HHS super-stub honeycomb cores with FSW facesheets, while the ratio limit moved from 0.7 to 1.0 according to the results of GB 50429-2007 and EN 1999-1-1: 2007. Specifically, the average values of *N*_GB_/*N*_exp/FE_, *N*_EC9_/*N*_exp/FE_ and *N*_AADM_/*N*_exp/FE_ of large-scale SHS and HHS super-stub honeycomb cores without FSW facesheets were respectively 0.68, 0.69 and 0.67, with great discrete results of COVs of 0.258, 0.256 and 0.294. More conservative results occurred for large-scale SHS and HHS super-stub honeycomb cores with FSW facesheets predicted from GB 50429-2007 and EN 1999-1-1: 2007, as listed in Table 6. The very conservative and discrete results showed that the current three specifications are not appropriate for predicting the out-of-plane compression resistances of large-scale SHS and HHS super-stub honeycomb cores without and with FSW facesheets due to the fact that the height of specimens is only 100 mm, almost equivalent to their cross-section depth, with greater restraint from top and bottom boundary conditions, and the negative influence of the HAZ induced by FSW on resistances is slight, leading to an improvement in the compression resistances of super-stub honeycomb cores compared with those of ordinary stub columns featured with a height larger than three times the cross-section depth.

### 4.5. Improved Design Approach

In order to improve the accuracy of predictions of out-of-plane compression resistances of 6061-T6 aluminium alloy large-scale SHS and HHS super-stub honeycomb cores without and with FSW facesheets, modifications to GB 50429-2007 are proposed. As displayed in Figure 13, the test and FE data points of single SHS and HHS super-stub cells nearly coincided with each other, while those of double and four SHSs super-stub cells, as well as those of double and four HHSs super-stub cells, generally overlapped with each other. Similar findings were also observed for SHS and HHS super-stub honeycomb cores with FSW facesheets, as shown in Figure 14. Based on these characteristics, systematic modifications to the cross-section effective thickness of specimens without and with FSW facesheets were proposed for each case, as given by Equations (9)–(13),

Single square or hexagon section cell without welds:

(9) $$t_{e}/t=\left\{\begin{array}{l} 1.0\;\;\;\;\;\;\;\;\;\;\;\;\lambda_{p}\leq 0.7\\ \frac{0.938}{\lambda_{p}^{0.219}}\;\;\;\;\;\;0.7<\lambda_{p}\leq 1.15\\ 1.018-0.094\lambda_{p}\;\;\;1.15<\lambda_{p}\leq 2.50 \end{array}\right.$$

Double or four square section cells without welds:

(10) $$t_{e}/t=\left\{\begin{array}{l} 1.0\;\;\;\;\;\;\;\;\;\;\lambda_{p}\leq 0.7\\ \frac{0.947}{\lambda_{p}^{0.151}}\;\;\;\;\;\;0.7<\lambda_{p}\leq 1.15\\ 1.024-0.084\lambda_{p}\;\;\;1.15<\lambda_{p}\leq 2.50 \end{array}\right.$$

Double or four hexagon section cells without welds:

(11) $$t_{e}/t=\left\{\begin{array}{l} 1.0\;\;\;\;\;\;\;\;\;\;\;\;\lambda_{p}\leq 0.7\\ \frac{0.971}{\lambda_{p}^{0.081}}\;\;\;\;\;\;0.7<\lambda_{p}\leq 1.15\\ 1.004-0.038\lambda_{p}\;\;\;1.15<\lambda_{p}\leq 2.50 \end{array}\right.$$

Single square or hexagon section cell with FSW facesheets:

(12) $$t_{e}/t=\left\{\begin{array}{l} 1.0\;\;\;\;\;\;\;\;\;\;\;\;\lambda_{p}\leq 0.7\\ \frac{0.884}{\lambda_{p}^{0.342}}\;\;\;\;\;\;0.7<\lambda_{p}\leq 1.40\\ 0.909-0.086\lambda_{p}\;\;\;1.40<\lambda_{p}\leq 2.50 \end{array}\right.$$

Double or four square section cells with FSW facesheets:

(13) $$t_{e}/t=\left\{\begin{array}{l} 1.0\;\;\;\;\;\;\;\;\;\;\;\lambda_{p}\leq 0.7\\ \frac{0.898}{\lambda_{p}^{0.295}}\;\;\;\;\;\;0.7<\lambda_{p}\leq 1.40\\ 0.929-0.083\lambda_{p}\;\;\;1.40<\lambda_{p}\leq 2.50 \end{array}\right.$$

Double or four hexagon section cells with FSW facesheets:

(14) $$t_{e}/t=\left\{\begin{array}{l} 1.0\;\;\;\;\;\;\;\;\;\;\;\lambda_{p}\leq 0.7\\ \frac{0.919}{\lambda_{p}^{0.227}}\;\;\;\;\;\;0.7<\lambda_{p}\leq 1.40\\ 0.913-0.044\lambda_{p}\;\;\;1.40<\lambda_{p}\leq 2.50 \end{array}\right.$$

**Figure 15:**
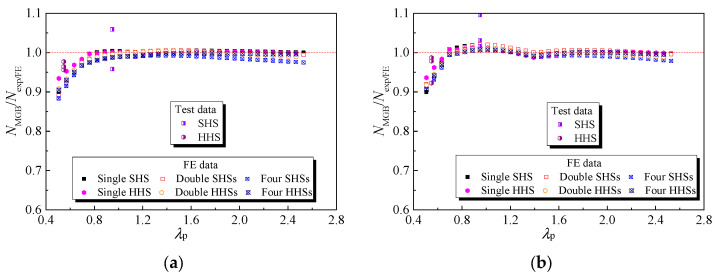
Comparisons of test and FE results with modified GB 50429: (**a**) Non-welded honeycomb specimens; (**b**) Honeycomb specimens with FSW facesheets.

It can be seen that three segment curves for obtaining the cross-section effective thickness were derived to replace the codified curves presented by Equation (2). Moreover, it is not necessary to classify cross-sections by comparing the width-to-thickness ratio of the plate with the codified limit and then determine the cross-section effective thickness as they fall within the slender range. Note that Equations (9)–(13) were restrained to the case that the height of specimens is 100 mm and the height of the HAZ is 10 mm with yielding and ultimate strength reduction of 0.68 and 0.8, respectively. The non-dimensional out-of-plane compression resistances of 6061-T6 aluminium alloy large-scale SHS and HHS super-stub honeycomb cores without and with FSW facesheets calculated by the modified GB 50429-2007 (*N*_MGB_/*N*_exp/FE_) are plotted against the non-dimensional slenderness of plate (*λ*_p_), as shown in Figure 15. Most test and FE data points were prone to be unity as *λ*_p_ > 0.75 and *λ*_p_ > 0.70, respectively, for honeycomb specimens without and with FSW facesheets. The relatively conservative results of specimens with smaller *λ*_p_ are presented in Figure 15, which was attributed to the strain hardening effect after the yielding of the cross-section. As listed in Table 7, the average values of *N*_MGB_/*N*_exp/FE_ for honeycomb specimens without and with FSW facesheets were both 0.99~1.00, with significantly reduced discrete results of COVs of 0.022–0.026 and 0.019–0.025, respectively, revealing that the modified design approach on the cross-section effective thickness yielded much more accurate and consistent out-of-plane compression resistance predictions compared to the current codified design provisions for 6061-T6 aluminium alloy large-scale SHS and HHS super-stub honeycomb cores without and with FSW facesheets.

## 5. Conclusions

The out-of-plane compression behaviour and resistances of 6061-T6 aluminium alloy large-scale SHS and HHS super-stub honeycomb cores without and with FSW facesheets were investigated and presented in this paper. The main conclusions can be drawn as follows:

(1) The non-welded and welded SHS honeycomb specimens failed by local buckling before cross-section yielding, while the non-welded and welded HHS honeycomb specimens failed by local buckling after cross-section yielding.
(2) The design rules set out in GB 50429-2007, EN 1999-1-1: 2007 and AADM-2015 generally yielded very conservative predictions for the out-of-plane compression resistances of aluminium alloy SHS and HHS super-stub honeycomb cores without and with FSW facesheets by about 30–37%, based on the experimental and parametric analysis results.
(3) Modifications to the cross-section effective thickness within the framework of GB 50429-2007 method, applicable to single, double and four SHS and HHS super-stub cells without and with FSW facesheets, were proposed and shown to provide much more accurate and consistent predictions than current design methods.
(4) Facesheets can be effectively fixed to the end of SHS and HHS honeycomb cores to form a sandwich configuration by FSW without failure occurring at the weld zone and with a strength reduction of less than 11% compared with non-welded specimens.

## Figures and Tables

**Table 1:** Measured dimensions of honeycomb specimens.

Specimen	*B* (mm)	*H* (mm)	*t* (mm)	*t_p_* (mm)	*A*_g_ (mm^2^)	*L* (mm)
S180-5-1	180.2	180.4	5.0	—	3585.8	100.4
S180-5-2	354.7	180.2	4.9	—	6318.4	101.0
S180-5-4	355.3	355.0	4.9	—	10,912.7	100.7
H104-5-1	104.1	104.4	5.1	—	3069.6	100.5
H104-5-2	103.7	103.8	5.0	—	5614.8	100.4
H104-5-4	104.3	104.2	4.8	—	9812.7	100.6
S180-5-1-FSW	179.6	180.1	5.1	2.0	3588.5	101.2
S180-5-2-FSW	355.2	180.3	5.0	1.9	6321.7	100.8
S180-5-4-FSW	355.2	355.2	5.0	2.0	10,919.4	99.2
H104-5-1-FSW	104.6	105.2	4.9	2.0	3061.7	100.5
H104-5-2-FSW	105.1	105.1	4.9	2.0	5611.4	100.6
H104-5-4-FSW	104.8	105.3	5.1	2.0	9821.2	100.4

**Table 2:** Measured material properties.

Category	Specimen	Thickness (mm)	*E*	*f*_0.2_	*f*_u_	*ε*_u_ (%)	*n*
			(GPa)	(MPa)	(MPa)		
Base aluminium alloy coupons	0-1	5.02	71,783	245.63	299.82	5.37	16.12
	0-2	4.93	67,276	250.11	302.92	5.60	16.89
	0-3	4.91	67,712	251.20	304.16	5.18	16.78
	SD	0.048	2030	2.41	1.83	0.17	0.34
	1-1	2.04	66,721	172.07	225.78	11.23	14.91
	1-2	2.07	67,626	171.65	225.09	10.67	14.75
	1-3	1.96	65,707	171.34	224.38	12.48	14.88
	SD	0.046	784	0.30	0.57	0.76	0.07
Welded aluminium alloy coupons	2-1	5.08	68,942	146.17	241.87	4.01	5.71
	2-2	5.04	68,615	153.51	240.30	4.12	5.89
	2-3	5.03	71,162	159.15	236.07	3.89	6.56
	SD	0.022	1131	5.31	2.45	0.09	0.37
	3-1	2.06	68,225	130.19	190.16	3.96	7.74
	3-2	2.01	67,188	131.79	169.42	2.43	9.42
	3-3	1.95	71,173	130.14	188.20	3.67	7.36
	SD	0.045	1688	0.77	9.35	0.66	0.90

Note: SD denotes the standard deviation.

**Table 3:** Experimental and numerical compression capacities of honeycomb specimens.

Specimen	*N*_exp_ (kN)	*N*_FE_ (kN)	*N*_FE_ /*N*_exp_	Specimen	*N*_exp_ (kN)	*N*_FE_ (kN)	*N*_FE_ /*N*_exp_
S180-5-1	806.66	849.50	1.05	H104-5-1	781.22	806.16	1.03
S180-5-1-FSW	804.62	762.26	0.95	H104-5-1-FSW	773.25	786.51	1.02
S180-5-2	1431.01	1521.37	1.06	H104-5-2	1462.03	1497.84	1.02
S180-5-2-FSW	1414.53	1366.81	0.97	H104-5-2-FSW	1428.72	1473.32	1.03
S180-5-4	2731.05	2645.16	0.97	H104-5-4	2534.97	2634.86	1.04
S180-5-4-FSW	2273.35	2374.61	1.04	H104-5-4-FSW	2650.53	2582.97	0.97
Mean							1.01
COV							0.037

**Table 4:** Dimensions of SHS honeycomb specimens for parametric analysis (*t* = 5, unit: mm).

*b*_f_/*t*	*B* × *H*	*b*_f_/*t*	*B* × *H*	*b*_f_/*t*	*B* × *H*
16	90 × 90	38	200 × 200	60	310 × 310
18	100 × 100	40	210 × 210	62	320 × 320
20	110 × 110	42	220 × 220	64	330 × 330
22	120 × 120	44	230 × 230	66	340 × 340
24	130 × 130	46	240 × 240	68	350 × 350
26	140 × 140	48	250 × 250	70	360 × 360
28	150 × 150	50	260 × 260	72	370 × 370
30	160 × 160	52	270 × 270	74	380 × 380
32	170 × 170	54	280 × 280	76	390 × 390
34	180 × 180	56	290 × 290	78	400 × 400
36	190 × 190	58	300 × 300	80	410 × 410

**Table 5:** Dimensions of HHS honeycomb specimens for parametric analysis (*t* = 5, unit: mm).

*b*_f_/*t*	*B* × *H*	*b*_f_/*t*	*B* × *H*	*b*_f_/*t*	*B* × *H*
16.05	86 × 86	38.05	196 × 196	60.05	306 × 306
18.05	96 × 96	40.05	206 × 206	62.05	316 × 316
20.05	106 × 106	42.05	216 × 216	64.05	326 × 326
22.05	116 × 116	44.05	226 × 226	66.05	336 × 336
24.05	126 × 126	46.05	236 × 236	68.05	346 × 346
26.05	136 × 136	48.05	246 × 246	70.05	356 × 356
28.05	146 × 146	50.05	256 × 256	72.05	366 × 366
30.05	156 × 156	52.05	266 × 266	74.05	376 × 376
32.05	166 × 166	54.05	276 × 276	76.05	386 × 386
34.05	176 × 176	56.05	286 × 286	78.05	396 × 396
36.05	186 × 186	58.05	296 × 296		

**Table 6:** Comparison of test/FE results with codified predicted strengths.

Non-welded honeycomb specimens			
Ratio	*N*_GB_/*N*_exp/FE_	*N*_EC9_/*N*_exp/FE_	*N*_AADM_/*N*_exp/FE_
Mean	0.68	0.69	0.67
COV	0.258	0.256	0.294
Honeycomb specimens with FSW facesheets			
Ratio	*N*_GB_/*N*_exp/FE_	*N*_EC9_/*N*_exp/FE_	*N*_AADM_/*N*_exp/FE_
Mean	0.63	0.64	0.70
COV	0.178	0.173	0.276

**Table 7:** Comparison of test/FE results with modified predicted strengths.

Non-welded honeycomb specimens			
Case	Single SHS or HHS	Double or four SHSs	Double or four HHSs
Ratio	*N*_MGB_/*N*_exp/FE_	*N*_MGB_/*N*_exp/FE_	*N*_MGB_/*N*_exp/FE_
Mean	1.00	0.99	0.99
COV	0.022	0.026	0.022
Honeycomb specimens with FSW facesheets			
Case	Single SHS or HHS	Double or four SHSs	Double or four HHSs
Ratio	*N*_MGB_/*N*_exp/FE_	*N*_MGB_/*N*_exp/FE_	*N*_MGB_/*N*_exp/FE_
Mean	1.00	1.00	0.99
COV	0.019	0.025	0.021

## Data Availability

Additional data can be obtained upon reasonable request from the corresponding author.
